# The relationship between physical activity, body fatness and metabolic syndrome in urban South African school teachers: The sympathetic activity and ambulatory blood pressure in Africans study

**DOI:** 10.4102/phcfm.v14i1.3133

**Published:** 2022-05-30

**Authors:** Tamrin Veldsman, Mariette Swanepoel, Johanna S. Brits, Makama A. Monyeki

**Affiliations:** 1 Physical Activity, Sport and Recreation Research Focus Area (PhASRec), Faculty of Health Sciences, North-West University, Potchefstroom, South Africa; 2 Department of Physiology, Faculty of Health Sciences, North-West University, Potchefstroom, South Africa

**Keywords:** body fatness, metabolic syndrome, physical activity, SABPA study, teacher

## Abstract

**Background:**

Globally, the prevalence of metabolic syndrome (MS) is rising because of increased levels of physical inactivity and obesity. In South Africa, information about teachers’ physical activity (PA), body fatness and MS is limited.

**Aim:**

To assess the relationship between PA, body fatness and MS in urban South African teachers.

**Setting:**

The study setting was in Dr Kenneth Kaunda District in the North West province of South Africa.

**Methods:**

A cross-sectional study was conducted using secondary data drawn from the sympathetic activity and ambulatory blood pressure in Africans (SABPA) study of 216 teachers (aged 25–65 years). Variables included anthropometry, biochemical measurements, objectively measured PA and lifestyle behaviours.

**Results:**

Twenty-nine percent of the total participants were classified with MS, with 46% in men compared to 13% in women; 33% were sedentary and 67% participated in light activity. A weak significant negative relationship was found between the mean 7-day awake metabolic equivalent of tasks (METs) and triglycerides (*r* = −0.29; *p* = 0.02) and gamma-glutamyl transferase (*r* = −0.25; *p* = 0.06), activity energy expenditure (*r* = −0.24; *p* = 0.06) and PA level (*r* = −0.23; *p* = 0.07). After adjusting for age, self-reported smoking and alcohol use or consumption, a weak significant negative relationship between mean 7-day awake METs and triglycerides (*r* = −0.28; *p* < 0.01) was observed.

**Conclusion:**

In the teachers with MS, only one MS marker (triglycerides) showed a negative association with PA. Physical activity could therefore be beneficial in the regulation of triglycerides. Participation in regular PA could be beneficial in the regulation of triglycerides. Focused PA interventions in school teachers that advocate the benefits of PA and healthy lifestyle choices to reduce dietary fat intake (and alcohol) are recommended.

## Background

The global incidence of metabolic syndrome (MS) is rising and becoming a clinical and public health concern.^1,2^ Persons diagnosed with MS are at risk for developing cardiovascular heart diseases (CVD) in the next 5–10 years, and MS will increase the risk of all-cause mortality by 1.5 times compared with apparently healthy individuals.^1,3^ Metabolic syndrome is referred to as a complex cluster of metabolic risk factors in the development of CVD and type two diabetes that includes elevated blood pressure (BP), triglyceride and total cholesterol levels, low high-density lipoprotein (HDL) levels and increased levels of central obesity.^1,4,5^

Globally, the prevalence of MS is estimated to be 17% – 25%.^6^ In urban South Africa, MS varies between 0% and 50% or higher, depending on the definition used to classify MS and the population studied.^6^ In a study conducted in an urban community in Free State Province, South Africa, 39.7% of participants were indicated to have three or more risk factors for the development of MS,^7^ and in Gauteng Province, South Africa, 29% of Africans were diagnosed with MS.^8^ The variance in the prevalence may be explained by differences in the MS diagnostic criteria.^9^ Regarding the different diagnostic criteria for MS,^10^ the most commonly used criteria include those of the World Health Organization (WHO), the International Diabetes Federation (IDF) and the National Cholesterol Education Program Adult Treatment Panel (NCEP ATP III). However, in 2009, the Joint Interim Statement (JIS) by the IDF, National Heart, Lung and Blood Institute, American Heart Association, World Heart Federation, International Atherosclerosis Society and International Association for the Study of Obesity proposed a harmonised definition of MS that considers the different waist circumferences (WCs) of different ethnic groups.^1^ This study used the JIS definition for MS to include ethnicity-specific cut-points for WC.

Physical inactivity (i.e. represents the non-achievement of physical activity [PA] guidelines) is a key risk factor in developing MS and leads to an increase in the risk of developing MS by 73%.^11,12^ The increasing prevalence of physical inactivity and obesity may lead to an increased global prevalence of MS.^1,9^ Physical inactivity is inversely associated with health outcomes.^13^ In addition, in both self-reported^14,15^ and objective data,^13,16^ low levels of PA are reported to be associated with MS. Abdominal obesity is a common sign in a person presenting with MS.^1^ Moderate and high levels of PA are related to a reduced risk of developing MS.^17^

With the increasing global prevalence of MS and the dire health consequences thereof, the risk factors of MS across the population need to be well understood.^13^

Teachers are considered to be physically inactive and spend most of their working time in sedentary or light energy-cost activities.^18^ In a South African study, 18.7% of teachers in Cape Town were at an increased risk of having a myocardial infarction or stroke within the next decade.^19^ More than half of the teachers were classified as either being overweight or obese.^19^ As a result of the working conditions of teachers that foster high levels of physical inactivity, the high prevalence of overweight and obesity amongst teachers and their high risk of developing a CVD,^18^ studies are needed to explore the prevalence of MS and the relationships between MS, PA and body fatness. This study aimed to assess the relationships between PA, body fatness and MS in urban South African teachers to determine if there is an association between being physically inactive and MS.

## Methods

### Study design

This cross-sectional study used secondary data from the SABPA prospective cohort study (*N* = 409).^20,21^ The initial SABPA study (phase 1) was conducted in 2008–2009; however, the follow-up data (phase 2), as was used in this study, was collected during 2011–2012.^20^ The SABPA study aims were to determine neural mechanistic pathways involved in emotional distress and vascular remodelling in urban South African teachers.^20^ In this study, seasonal changes were avoided and extensive clinical measurements were collected in well-controlled settings.^20^ In the larger study, sociodemographic information, behavioural lifestyle habits and biological variables, psychosocial battery of tests, mental stress responses and target end organ damage in the brain, heart, kidney, blood vessels and retina were collected.^20^

### Study population and sampling

Phase 1 of the SABPA study used convenient sampling; all teachers in the Dr Kenneth Kaunda District were recruited to form part of the SABPA study.^20^ The study sample comprised selected urbanised South African teachers in Dr Kenneth Kaunda District in the North West province of South Africa. The population was chosen to obtain a similar sample from a comparable socioeconomic class. Participants were aged between 25 and 65 years. Exclusion criteria used for phase 1 and phase 2 included if the participants were pregnant, lactating or using α- or β-blockers; if they had psychotropic substance dependence; if they had donated blood or been vaccinated in the previous 3 months or if they had an ear temperature above 37 °C. Only participants who wore the ActiHeart for a full seven days with < 40 min of inappropriate ActiHeart contact (*N* = 216) were included. Detailed methodology of the SABPA study has been published elsewhere.^20^ Power analyses were performed for the SABPA cohort study using previous studies. Ambulatory autonomic dysfunction and cortisol were variables used to obtain effect sizes based on differences in biological profiles and genotyping the hypothalamic–pituitary–adrenal (HPA) axis. Ultimately, a sample size at a statistical power of 0.8 with a level of significance set at 0.05 yielded a total of 50–416 that explained the biological differences and detection of single nucleotide polymorphisms (SNPs).^20^

Ambulatory dysfunction^22^ and cortisol data^23,24^ were used to obtain relative effect sizes based on differences in biological profiles and genotyping HPA axis variation. This resulted in sample sizes of 50–416 to explain biological differences and to detect SNPs with a statistical power of 0.8 and a level of significance of 0.05.^22, 23,24^

This study formed a part of the SABPA cohort study by virtue of the group size of 50–416 selected participants who met the inclusion criteria.

### Data collection

All data used for this study were collected during the initial SABPA cohort study.^20^

#### Anthropometric measurements

Anthropometric data were collected in the original SABPA study^20^ by a level 2 kinanthropometrist who measured participants’ height (cm), weight (kg) and WC (cm) in minimal clothing, according to the International Society for the Advancement of Kinanthropometry (ISAK) guidelines.^25^ The body mass index (BMI) of participants was calculated by dividing body weight in kilograms by height in metres squared.^25^ Waist-to-height ratio (WHtR) was calculated by dividing WC by height in cm.^26^ Intra- and inter-observer variability were < 10%.

#### Biochemical measurements

A sterile winged infusion set was used to obtain blood samples from the antebrachial vein branches of the right arm by a registered nurse before 09:00, handled according to standardised procedures and stored at –80 °C until blood analyses were performed. All blood samples were obtained from never-thawed serum, plasma and citrate samples. Sodium fluoride (NaF), HDL, gamma-glutamyl transferase (GGT) and triglycerides were measured and analysed using the Konelab 20i sequential multiple analyser computer (SMAC) (Thermo Scientific, Vantaa, Finland) and Unicel DXC 800 (Beckman and Coulter, Germany) at accredited independent laboratories as described in SABPA study.^20^ Inter- and intra-variability values accrued to < 5%. These values were determined with an enzyme rated method (Unicel DXC 800, Beckman and Coulter, Germany); homogeneous immunoassay (Modular Roche Automized systems, Basel, Switzerland) and a particle enhanced turbidimetric assay (Cobas Integra 400 plus, Roche, Basel, Switzerland), respectively.

#### Lifestyle behaviours

Lifestyle behavioural factors used in the study were based on available data from closed-ended questions (yes or no) to determine smoking habits and alcohol usage of the participants. The lifestyle questionnaire was conducted in private at the research facility of the North-West University. The questionnaire was explained by trained research personnel to each participant before the completion thereof. The participants completed the questionnaire on a hard copy and were encouraged to ask questions if they did not understand a specific question. Secondary smoking was included in the participants’ smoking habits. Objectively measured GGT was also used as a measure of alcohol usage.

#### Blood pressure

Professionally trained personnel (i.e. nurse or physiologist) measured resting BP in a semi-recumbent position with a sphygmomanometer (1.3M^TM^ Littman^®^ II S.E. Stethoscope 2205, Reister CE 0124, No. 1010–108 Diplomat-presameter^®^, Germany) on the non-dominant arm using the Rica/Rocci Korotkoff method. After 5 min, the BP measurement was repeated and the second measurement value was used for the MS criteria for BP.^1^

#### Metabolic syndrome

The JIS classification was used to determine the prevalence of MS. Participants were classified as presenting with MS when three or more risk factors for MS were present.^1^ The JIS classification takes ethnicity-specific cut-points into consideration. Ethnicity-specific waist circumference values were indicated as a risk at ≥ 94 cm and ≥ 80 cm for Caucasian men and women, respectively,^1^ and for Africans WC was indicated as a risk at ≥ 90 cm for men and ≥ 98 cm for women.^27^ Other risk factors of MS included HDL in men ≤ 1.0 mmol/L and in women ≤ 1.3 mmol/L, triglycerides ≥ 1.70 mmol/L, fasting glucose ≥ 5.60 mmol/L, systolic BP (SBP) ≥ 130 mmHg and diastolic BP (DBP) ≥ 85 mmHg and being on hypertensive treatment.^1^

#### Physical activity

The PA of participants was measured over seven consecutive days using an ActiHeart (GNO/67703, CamNtech Ltd., Cambridgeshire, United Kingdom) combined heart rate and accelerometer device. A registered nurse performed a resting 12-lead electrocardiogram (Norav Medical Ltd PC 1200, software version 5.030, Kiryat Bialik, Israel). From the electrocardiogram, the participants’ resting heart rate was captured. Sleep heart rate was calculated as resting heart rate minus 10 beats per minute. Sleep heart rate was required by the ActiHeart programme when the device was fitted to the participants.

The individual step test calibration of the ActiHeart could not be performed because of the participants’ high cardiovascular risk profile and time constraints during data collection, as there were a large number of participants; however, a biokineticist (clinical exercise physiologist) trained in PA behaviour thoroughly questioned the participants regarding their PA behaviour in order to choose a PA level to programme the ActiHeart. Heart rate, the MET (1 MET regarded as being asleep) and activity level were used to differentiate sleeping time from time awake. When a gradual decrease in heart rate was measured during the evenings (with 15 or more epochs) to below average heart rate in a sedentary awake-time sample period, and the activity level was equal to zero, the participant was considered asleep. The end of sleeping time was identified as an immediate increase in the participant’s heart rate of more than 10–20 beats per minute relative to the given sleeping heart rate, together with an increased MET and activity level. The distribution of PA intensity was expressed as sedentary (< 1.5 METs), time spent in light PA (1.5–3 METs) and time spent in moderate-to-vigorous PA (> 3 METs).^28^ The total energy expenditure (TEE) of an individual was expressed in kilocalories (kcal) and was composed of the resting energy expenditure (REE), diet-induced energy expenditure (DEE) and activity-induced energy expenditure (AEE). Physical activity level (PAL) was calculated as TEE/REE.^29^

### Data analysis

Data were analysed using the Statistical Package for Social Sciences (SPSS) version 26 (Inc., Chicago, IL, United States [US]). The data distribution was evaluated using histograms and quantile-quantile plots. Descriptive statistics (means, standard deviations) were calculated for PA, body fatness (i.e. BMI, WC and WHtR) and MS. Frequencies for percentages were calculated. The independent t-test for normally distributed data were used to determine the differences amongst groups. Chi-square was used to determine significant differences between the expected frequencies and the observed frequencies in one or more categories. For data that were not normally distributed, the Mann–Whitney U test was used to compare significant differences between two independent groups when the dependent variable was either ordinal or continuous. To study the relationship between PA, body fatness (i.e. BMI, WC and WHtR) and MS, correlation coefficients were calculated and partial correlation controlled for age, sex and lifestyle behaviour (smoking and alcohol drinking [GGT]) for the entire group and for those with MS and without MS. Correlation coefficient values were classified as follows: between < 0.10 and 0.29 indicates a weak or small correlation, 0.30–0.5 indicates a moderate correlation and ≥ 0.50 indicates a large correlation. Statistical significance was set at *p* ≤ 0.05.^30^

### Ethical considerations

Ethical clearance was obtained from the Health Research Ethics Committee (HREC) of North-West University (NWU-00036-07-S6), and the study conformed to the ethical principles outlined in the Declaration of Helsinki (revised 2004). Permission for this study was obtained from the North West Department of Education, as well as the South African Democratic Teachers’ Union. Before recruitment, participants were informed about the study and assistance was available to participants who preferred to receive the information in their home language. Participants were allowed to refuse data collection and were not contacted again. The anonymity and confidentiality of participants were assured throughout the study by allocating a coded number to each participant.

## Results

**FIGURE 1:**
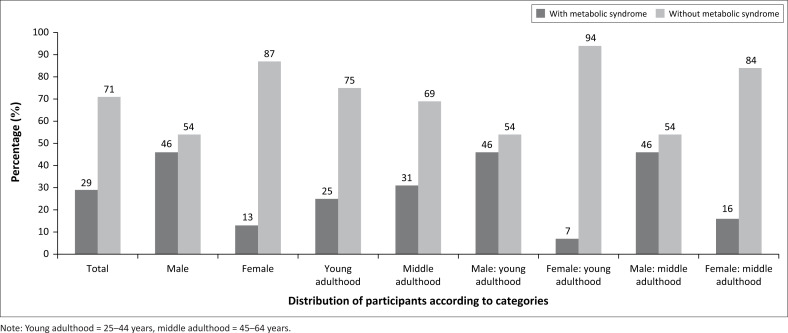
The prevalence of metabolic syndrome amongst teachers.

From a total of 216 teachers (males; *n* = 104; females; *n* =112), 29% were classified with MS, with almost half of the male teachers (46%) classified with MS (Figure 1). After dividing the participants into age groups according to the guidelines suggested by Statistics: Provisional Guidelines on Standard International Age Classification of 1982 (UNDESA),^31^ more middle adulthood (45–64 years) teachers (31%) than young adulthood (25–44 years) teachers (25%) were found to present with MS. In addition, the frequency distribution of the participants according to age classification were: young adulthood 45–64 years 57, 26%; middle adulthood 159, 74%; young adulthood men 45–64 years 26, 25%; middle adulthood 78, 75%; young adulthood women 45–64 years 31, 28%; middle adulthood 81, 82%. Almost half of the males in both age groups (46%) presented with MS.

**FIGURE 2:**
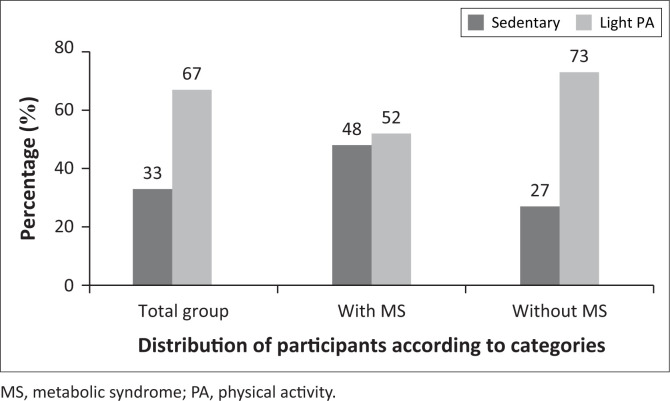
The prevalence of sedentary and light physical activity amongst teachers with and without metabolic syndrome.

Figure 2 depicts the PA amongst the entire group of teachers and according to classification as presenting with or without MS. In the entire group of teachers, 33% were sedentary and 67% were classified as lightly active. In the teachers with MS, almost half (48%) were classified as sedentary and 52% were classified as participating in light PA. In persons who did not present with MS, 73% of the participants were considered to be lightly active.

**FIGURE 3:**
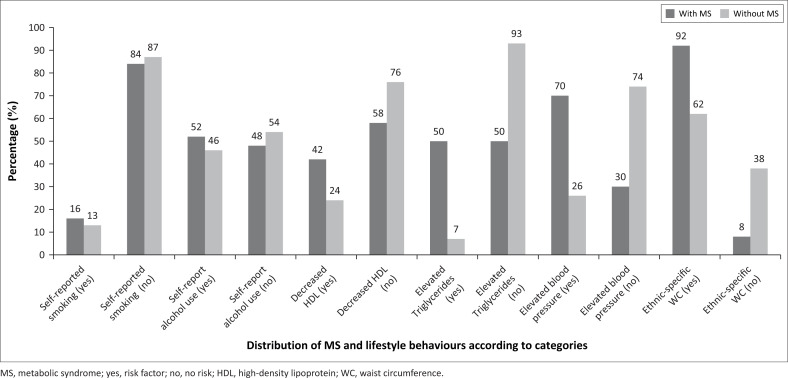
Distribution of prevalence of metabolic risk factors and lifestyle behaviours amongst teachers presenting with and without metabolic syndrome.

Figure 3 indicates the prevalence of metabolic risk factors and lifestyle behaviours amongst teachers presenting with MS and without MS. Amongst the teachers presenting with MS, the risk factor with the highest prevalence (92%, 58/63) was having a WC larger than the ethnicity-specific cut-points developed by Prinsloo and colleagues.^27^ The second highest prevalent metabolic risk factor was having BP equal to or higher than 130/85 mmHg (70%).^1^ More than half (52%) of the participants reported drinking alcohol. The prevalence of self-reported smoking was 16%. Elevated triglyceride levels were present in half of the teachers (50%) and decreased HDL was present in almost half (42%) of the teachers. Furthermore, the results showed that HDL concentrations in teachers presenting with MS were significantly lower (Mann–Whitney U: 3852.00, *p* = 0.008) than in those without MS. Also, elevated triglycerides and BP were both significantly higher in teachers presenting with MS (Mann–Whitney U: 2681.50 and 2349.00, *p* < 0.001) than in those without MS. The teachers classified in the MS category (*n* = 58) had significantly higher WC (Mann–Whitney U, 3375.33, *p* < 0.001) than those without MS (*n* = 5).

Table 1 depicts the characteristics of the teachers according to diagnosis of MS. Teachers with MS were significantly taller (*p* < 0.002; 172.48 ± 9.89 cm vs 167.73 ± 10.00 cm) and heavier (*p* < 0.001; 93.91 ± 19.43 kg vs 79.42 ± 18.13 kg) than those without MS and had significantly greater BMI (*p* = 0.001; 31.71 ± 6.92 kg/m^2^ vs 28.39 ± 6.06 kg/m^2^), WC (106.59 ± 15.28 cm vs 92.20 ± 14.23 cm) and WHtR (*p* < 0.001; 0.62 ± 0.10 vs 0.55 ± 0.08). Gamma-glutamyl transferase in teachers with MS (71.83 ± 87.40 U/L) was more than double than that of the teachers without MS (32.50 ± 34.36 U/L). Total cholesterol (*p* < 0.001; 4.92 ± 1.14 mmol/L vs 4.27 ± 0.91 mmol/L), triglyceride (*p* < 0.001; 1.80 ± 1.10 mmol/L vs. 1.02 ± 0.50 mmol/L) and glucose values (6.34 ± 2.92 mmol/L vs. 4.48 ± 0.74 mmol/L) were significantly higher in teachers with MS than those without MS. Systolic BP (*p* < 0.001; 141.98 ± 15.14 mmHg vs. 123.80 ± 16.80 mmHg) and DBP (*p* < 0.001; 93.95 ± 8.73 vs 83.04 ± 10.74 mmHg) were also significantly higher in teachers with MS.

**TABLE 1:** Descriptive characteristics of participants presenting with metabolic syndrome and participants without metabolic syndrome.

Variables	MS	*n*	Mean ± s.d.	*t*	*df*	*p*
Age (years)	Yes	63	51.25 ± 8.11	1.783	214	0.070
	No	153	49.01 ± 8.50			
Mean 7-day awake METs	Yes	63	1.85 ± 0.70	−1.403	214	0.160
	No	153	2.00 ± 0.68			
AEE (kcal/week)	Yes	63	1183.89 ± 998.66	−0.619	214	0.530
	No	153	1282.03 ± 1083.19			
TEE (kcal/week)	Yes	63	3291.41 ± 1188.29	0.201	214	0.840
	No	153	3250.99 ± 1400.56			
PAL	Yes	63	1.99 ± 0.68	−0.878	214	0.380
	No	153	2.08 ± 0.63			
Height (cm)	Yes	63	172.48 ± 9.89	3.181	214	0.002*
	No	153	167.73 ± 10.00			
Weight (kg)	Yes	63	93.91 ± 19.43	5.224	214	< 0.001*
	No	153	79.42± 18.13			
BMI (kg/m^2^)	Yes	63	31.71 ± 6.92	3.496	214	0.001*
	No	153	28.39 ± 6.06			
WC (cm)	Yes	63	106.59 ± 15.28	6.608	214	< 0.001*
	No	153	92.20 ± 14.23			
GGT (U/L)	Yes	61	71.83 ± 87.40	4.724	213	< 0.001*
	No	152	32.50 ± 34.36			
TC (mmol/L)	Yes	62	4.92 ± 1.14	4.724	213	< 0.001*
	No	153	4.27 ± 0.91			
HDL (mmol/L)	Yes	62	0.99 ± 0.34	−1.360	212	0.170
	No	152	1.06 ± 0.33			
Glucose (mmol/L)	Yes	62	6.34 ± 2.92	7.320	212	< 0.001*
	No	152	4.48 ± 0.74			
TG (mmol/L)	Yes	62	1.80 ± 1.10	7.083	213	< 0.001*
	No	153	1.02 ± 0.50			
SBP (mmHg)	Yes	63	141.98 ± 15.14	7.432	214	< 0.001*
	No	153	123.80 ± 16.80			
DBP (mmHg)	YES	63	93.95 ± 8.73	7.144	214	< 0.001*
	No	153	83.04 ± 10.74			
WHtR	Yes	63	0.62 ± 0.10	5.332	214	< 0.001*
	No	153	0.55 ± 0.08			

Note: Yes, participants with metabolic syndrome (MS); No, participants without metabolic syndrome

AEE, activity energy expenditure; BMI, body mass index; DBP, diastolic blood pressure; GGT, gamma-glutamyl transferase; HDL, high-density lipoprotein; METs, metabolic equivalent of task; MS, metabolic syndrome; PAL, physical activity level; SBP, systolic blood pressure; TC, total cholesterol; TEE, total energy expenditure; TG, triglycerides; WC, waist circumference; WHtR, waist-to-height ratio; s.d., standard deviation; *df*, degree of freedom.

* , The level of significance is set as *p* ≤ 0.05.

Table 2 depicts the relationship between PA, body fatness (i.e. BMI, WC and WHtR) and metabolic risk factors in the total group of teachers. In the relationship between PA and the anthropometric variables (BMI, WC and WHtR), a weak significant positive relationship was found between TEE and WC (*r* = 0.17; *p* = 0.02). Regarding the relationship between PA and the metabolic risk factors, AEE (*r* = −0.19; *p* = 0.01) and PAL (*r* = −0.16; *p* = 0.02) showed a weak significant negative relationship with GGT. Furthermore, an inconclusive weak borderline negative relationship was found between PAL and triglycerides (*r* = −0.13; *p* = 0.06). Regarding the relationship between the anthropometric variables and the metabolic risk factors, weak significant positive relationships were found between BMI and GGT (*r* = 0.28, *p* < 0.001); triglycerides (*r* = 0.25; *p* < 0.001); and DBP (*r* = 0.29; *p* < 0.001; and moderate significant relationship between glucose (*r* = 0.35; *p* < 0.001); SBP (*r* = 0.33; *p* < 0.001). Moderate significant positive relationships were observed between WC and GGT (*r* = 0.38; *p* < 0.001); glucose (*r* = 0.44; *p* < 0.001); triglycerides (*r* = 0.40; *p* < 0.001); SBP (*r* = 0.43; *p* <0.001) and DBP (*r* = 0.41; *p* < 0.001). Between WHtR and the metabolic risk factors, moderate significant positive relationships were observed with GGT (*r* = 0.36; *p* < 0.001); glucose (*r* = 0.43; *p* < 0.001); triglycerides (*r* = 0.33; *p* < 0.001); SBP (*r* = 0.40; *p* < 0.001) and DBP (*r* = 0.35; *p* < 0.001).

**TABLE 2:** The relationship between physical activity, body fatness and metabolic risk factors in a cohort of urban South African teachers.

Variables	BMI (kg/m^2^)	WC (cm)	Smoking	Alcohol use	GGT (U/L)	Glucose (mmol/L)	TC (mmol/L)	TG (mmol/L)	SBP (mmHg)	DBP (mmHg)	WHtR
**AEE (kcal/week)**											
*r*	−0.040	−0.020	0.010	0.060	−0.190**	−0.100	0.070	−0.110	−0.070	−0.080	−0.080
*p*	0.530	0.730	0.890	0.380	0.010	0.120	0.310	0.120	0.330	0.240	0.260
**TEE (kcal/week)**											
*r*	0.090	0.170*	−0.050	−0.010	−0.070	−0.003	0.070	0.030	0.030	0.040	0.060
*p*	0.210	0.020	0.440	0.890	0.310	0.960	0.290	0.720	0.710	0.600	0.400
**PAL**											
*r*	0.100	0.001	−0.040	0.020	−0.160*	−0.090	−0.050	−0.130***	−0.070	−0.070	0.050
*p*	0.130	0.980	0.600	0.750	0.020	0.490	0.510	0.060	0.340	0.340	0.510
**BMI (kg/m^2^)**											
*r*	-	0.840**	0.020	−0.030	0.280**	0.350**	0.080	0.250**	0.330**	0.290**	0.910**
*p*	-	< 0.001	0.770	0.700	< 0.001	< 0.001	0.270	< 0.001	< 0.001	< 0.001	< 0.001
**WC (cm)**											
*r*	0.840**	-	−0.060	−0.110	0.380**	0.440**	0.110	0.400**	0.430**	0.410**	0.920**
*p*	< 0.001	-	0.400	0.100	< 0.001	< 0.001	0.110	< 0.001	< 0.001	< 0.001	< 0.001
**Smoking**											
*r*	0.020	−0.060	-	0.180**	−0.180**	−0.040	−0.190**	−0.240**	−0.060	−0.080	−0.010
*p*	0.770	0.400	-	0.010	0.010	0.540	< 0.001	< 0.001	0.420	0.220	0.880
**Alcohol use**											
*r*	−0.030	−0.110	0.020**	-	−0.290**	−0.140b	0.010	−0.210**	−0.100	−0.120	−0.030
*p*	0.700	0.100	0.010	-	< 0.001	0.050	0.890	< 0.001	0.150	0.090	0.620
**GGT (U/L)**											
*r*	0.280**	0.380**	−0.180**	−0.290**	-	0.540**	0.210**	0.530**	0.380**	0.400**	0.360**
*p*	< 0.001	< 0.001	0.010	< 0.001	-	< 0.001	< 0.001	< 0.001	< 0.001	< 0.001	< 0.001
**Glucose (mmol/L)**											
*r*	0.350**	0.440**	−0.040	−0.140*	0.540**	-	0.210**	0.460**	0.400**	0.380**	0.430**
*p*	< 0.001	< 0.001	0.540	0.050	< 0.001	-	< 0.001	< 0.001	< 0.001	< 0.001	< 0.001
**TC (mmol/L)**											
*r*	0.080	0.110	−0.190**	0.010	0.210**	0.210**	-	0.370**	0.200**	0.180**	0.130*
*p*	0.270	0.110	< 0.001	0.890	< 0.001	< 0.001	-	< 0.001	< 0.001	0.010	0.050
**TG (mmol/L)**											
*r*	0.250**	0.400**	−0.240**	−0.210**	0.530**	0.460**	0.370**	-	0.280**	0.250**	0.330**
*p*	< 0.001	< 0.001	< 0.001	< 0.001	< 0.001	< 0.001	< 0.001	-	< 0.001	< 0.001	< 0.001
**SBP (mmHg)**											
*r*	0.330**	0.430**	−0.060	−0.100	0.380**	0.400**	0.200**	0.280**	-	0.840**	0.400**
*p*	< 0.001	< 0.001	0.420	0.150	< 0.001	< 0.001	< 0.001	< 0.001	-	< 0.001	< 0.001
**DBP (mmHg)**											
*r*	0.290**	0.410**	−0.080	−0.120	0.000**	0.380**	0.020**	0.250**	0.840**	-	0.350**
*p*	< 0.001	< 0.001	0.220	0.090	< 0.001	< 0.001	0.010	< 0.001	< 0.001	-	< 0.001
**WHtR**											
*r*	0.910**	0.920**	−0.010	−0.030	0.360**	0.430**	0.130*	0.330**	0.400**	0.350**	-
*p*	< 0.001	< 0.001	0.880	0.620	< 0.001	< 0.001	0.050	< 0.001	< 0.001	< 0.001	-

AEE, activity energy expenditure; BMI, body mass index; DBP, diastolic blood pressure; GGT, gamma-glutamyl transferase; PAL, physical activity level; SBP, systolic blood pressure; TC, total cholesterol; TEE, total energy expenditure; TG, triglycerides; WC, waist circumference; WHtR, waist-to-height ratio.

* , Correlation is significant at 0.001.

** , Correlation is significant at 0.01.

*** , border significance.

Table 3 depicts the Spearman’s correlation matrix (*rho*) for anthropometric variables and metabolic risk factors according to teachers presenting with MS and teachers without MS. Regarding the relationship between PA and the metabolic risk factors in teachers with MS, a weak significant negative relationship between mean 7-day awake METs and triglycerides (*r* = −0.29; *p* = 0.02) was found which was not present in those without MS. An inconclusive weak borderline significant negative relationship was found between GGT and mean 7-day awake METs (*r* = −0.25; *p* = 0.06); AEE (*r* = −0.24; *p* = 0.06) and TEE (*r* = −0.23; *p* = 0.07). However, in teachers without MS, only a weak significant negative relationship between mean 7-day awake METs and GGT (*r* = −20; *p* = 0.02) was observed. An inconclusive weak borderline significant positive association between AEE and self-reported smoking (*r* = 0.23; *p* = 0.07) was found in teachers with MS, which was not present in those without MS.

**TABLE 3:** Spearman correlation *rho* for anthropometric variables and metabolic syndrome variables according to teachers presenting with metabolic syndrome and without metabolic syndrome.

Variables	BMI (kg/m2)	WC (cm)	Smoking	Alcohol use	TC (mmol/L)	TG (mmol/L)	GGT (U/L)	SBP (mmHg)	DBP (mmHg)	WHtR
**MS participants (*n* = 62)**										
**Age group**										
*r*	−0.16	−0.07	0.19	0.13	−0.02	−0.16	−0.23	0.28*	0.01	−0.04
*p*	0.20	0.61	0.15	0.32	0.87	0.20	0.07	0.03	0.95	0.77
**Mean 7-day awake METS**										
*r*	0.10	−0.01	0.10	0.14	−0.19	−0.29*	−0.25***	−0.07	0.02	0.05
*p*	0.44	0.96	0.43	0.27	0.13	0.02	0.06	0.57	0.86	0.70
**AEE (kcal/week)**										
*r*	0.07	0.01	0.23***	0.13	0.07	−0.21	−0.24***	0.05	−0.09	0.05
*p*	0.59	0.92	0.07	0.31	0.59	0.10	0.06	0.71	0.50	0.72
**TEE (kcal/week)**										
*r*	0.16	0.13	0.17	0.11	−0.01	−0.19	−0.23***	−0.04	−0.16	0.08
*p*	0.23	0.33	0.17	0.41	0.95	0.14	0.07	0.74	0.20	0.55
**PAL**										
*r*	0.16	0.05	0.05	0.06	−0.12	−0.20	−0.18	0.04	0.11	0.15
*p*	0.22	0.67	0.69	0.65	0.35	0.12	0.17	0.78	0.40	0.23
**Non-MS participants (*n* = 153)**										
**Age group**										
*r*	−0.09	0.000	0.06	0.09	0.22**	0.04	−0.07	0.09	0.02	−0.03
*p*	0.30	0.99	0.47	0.28	0.01	0.59	0.43	0.27	0.86	0.70
**Mean 7-day awake METS**										
*r*	0.03	0.01	−0.10	−0.01	−0.02	−0.04	−0.20*	−0.15***	−0.13	−0.03
*p*	0.67	0.88	0.21	0.91	0.86	0.61	0.02	0.07	0.11	0.75
**AEE (kcal/week)**										
*r*	−0.05	0.02	−0.11	0.01	0.08	−0.02	−0.14	−0.04	−0.03	−0.09
*p*	0.51	0.79	0.18	0.87	0.31	0.82	0.09	0.61	0.74	0.29
**TEE (kcal/week)**										
*r*	0.08	0.22**	−0.16	−0.07	0.09	0.12	−0.01	0.06	0.11	0.07
*p*	0.33	0.01	0.06	0.42	0.27	0.14	0.94	0.44	0.17	0.42
**PAL**										
*r*	0.13	0.05	−0.09	−0.02	−0.001	−0.03	−0.08	−0.04	−0.04	0.07
*p*	0.12	0.53	0.28	0.85	0.99	0.74	0.35	0.59	0.62	0.43

Note: Age groups: young adulthood = 25–44 years, middle adulthood = 45–64 years.

AEE, activity energy expenditure; BMI, body mass index; DBP, diastolic blood pressure; GGT, gamma-glutamyl transferase; PAL, physical activity level; SBP, systolic blood pressure; MS, metabolic syndrome; TC, total cholesterol; TEE, total energy expenditure; TG, triglycerides; WC, waist circumference; WHtR, waist-to-height ratio.

* , The level of significance is set as *p* ≤ 0.05.

** , The level of significance is set as *p* ≤ 0.01.

*** , Indicates borderline significance.

Table 4 depicts the correlation coefficients (adjusted for age group, sex, self-reported smoking and alcohol usage) of the entire group of participants. Regarding the relationship between PA measurements and the metabolic risk factors, weak significant negative associations were found between mean 7-day awake METs and GGT (*r* = −0.19; *p* = 0.01) and triglycerides (*r* = −0.19; *p* = 0.01). In addition, TEE was weakly and negatively related with GGT (*r* = −0.17; *p* = 0.02) and triglycerides (*r* = −0.18; *p* = 0.01). Also, PAL was weakly negatively associated with triglycerides (*r* = −0.15; *p* = 0.03). An inconclusive weak borderline significant relationship was found between mean 7-day awake METs and total cholesterol (*r* = −0.13; *p* = 0.06). A weak significant negative relationship was found between GGT and AEE (*r* = −0.18; *p* = 0.01) and AEE and triglycerides (*r* = −0.20; *p* = 0.01). Weak significant negative relationships were found between TEE and GGT (*r* = −0.17; *p* = 0.02) and TEE and triglycerides (*r* = −0.18; *p* = 0.01). Regarding the relationship between the anthropometric variables and the metabolic risk factors, a weak significant positive relationship between WC and triglycerides (*r* = 0.16; *p* = 0.02) was found. Amongst the relationships with WHtR, a weak significant positive relationship with cholesterol (*r* = 0.17; *p* = 0.01) and triglycerides (*r* = 0.21; *p* < 0.001) was found. An inconclusive weak borderline significant association was also found between WHtR and GGT (*r* =0.13; *p* = 0.06). Furthermore, inconclusive weak borderline significant relationships were found between BMI and triglycerides (*r* = 0.13; *p* = 0.07), and cholesterol (*r* = 0.13; *p* = 0.06).

**TABLE 4:** Correlation coefficients adjusted for age group, sex, self-reported smoking and alcohol usage of the total group of teachers.

Variables	Mean 7-day awake METs	AEE (kcal/week)	TEE (kcal/week)	PAL	BMI (kg/m^2^)	WC (cm)	GGT (U/L)	Total Chol (mmol/L)	TG (mmol/L)	WHtR
**Mean 7-day awake METs**										
*r*	-	0.650*	0.580*	0.550*	0.040	0.010	−0.190*	−0.130**	−0.190*	−0.030
*p*	-	< 0.001	< 0.001	< 0.001	0.630	0.910	0.010	0.060	0.010	0.630
**AEE (kcal/week)**										
*r*	0.650*	-	0.960*	0.600*	0.004	−0.010	−0.180*	0.010	−0.200*	−0.040
*p*	< 0.001	-	< 0.001	< 0.001	0.960	0.940	0.010	0.870	0.010	0.590
**TEE (kcal/week)**										
*r*	0.580*	0.960*	-	0.530*	0.070	0.060	−0.170*	0.020	−0.180*	0.020
*p*	< 0.001	< 0.001	-	< 0.001	0.290	0.390	0.020	0.740	0.010	0.710
**PAL**										
*r*	0.550*	0.600*	0.530*	-	0.110	0.060	−0.120	−0.090	−0.150*	0.060
*p*	< 0.001	< 0.001	< 0.001	-	0.120	0.370	0.080**	0.200	0.030	0.400
**BMI (kg/m^2^)**										
*r*	0.040	0.004	0.070	0.110	-	0.890-*	0.060	0.130**	0.130**	0.900*
*p*	0.630	0.960	0.290	0.120	-	< 0.001	0.430	0.060	0.070	< 0.001
**WC (cm)**										
*r*	0.010	−0.010	0.060	0.060	0.890*	-	0.040	0.120	0.160*	0.960*
*p*	0.910	0.940	0.390	0.370	< 0.001	-	0.610	0.080	0.020	< 0.001
**GGT (U/L)**										
*r*	−0.190*	−0.180*	−0.170*	−0.120**	0.060	0.040	-	0.180*	0.220*	0.130**
*p*	0.010	0.010	0.020	0.080	0.430	0.610	-	0.010	0.001	0.060
**Total** chol **(mmol/L)**										
*r*	−0.130**	0.010	0.020	−0.090	0.130**	0.120	0.180*	-	0.360*	0.170*
*p*	0.060	0.870	0.740	0.200	0.060	0.080	0.010	-	< 0.001	0.010
**TG (mmol/L)**										
*r*	−0.190*	−0.200*	−0.180*	−0.150*	0.130**	0.160*	0.220*	0.360*	-	0.210*
*p*	0.010	0.010	0.010	0.030	0.070	0.020	0.001	< 0.001	-	< 0.001
**WHtR**										
*r*	−0.030	−0.040	0.020	0.060	0.900*	0.960*	0.130**	0.170*	0.210*	-
*p*	0.630	0.590	0.710	0.400	< 0.001	< 0.001	0.060	0.010	< 0.001	-

AEE, activity energy expenditure; Chol, cholesterol; GGT, gamma-glutamyl transferase; PAL, physical activity level; TEE, total energy expenditure; TG, triglycerides; WC, waist circumference; WHtR, waist-to-height ratio.

* , The level of significance is set as *p* ≤ 0.05.

** , Borderline significance.

Table 5 depicts the correlation coefficients adjusted for age group, sex, self-reported smoking and alcohol usage according to teachers with MS and without MS. Regarding the relationship between PA and metabolic risk factors amongst teachers with MS, a weak significant negative association was found between mean 7-day awake METs and triglycerides (*r* = −0.28; *p* = 0.03) which was not present in teachers without MS. In addition, a weak significant negative relationship was found between TEE and triglycerides (*r* = −0.26; *p* = 0.05), whilst an inconclusive weak borderline significant negative association was found between PAL and triglycerides (*r* = −0.24; *p* = 0.07). No statistically significant relationships were found between anthropometric variables and metabolic risk factors in teachers with MS. However, in teachers without MS, an inconclusive weak borderline significant negative association was found between GGT and mean 7-day awake METs (*r* = −0.16; *p* = 0.06). Triglycerides was moderately and positively associated with WC (*r* = 0.34; *p* < 0.001) and WHtR (*r* = 0.36; *p* < 0.001) in teachers without MS.

**TABLE 5:** Correlation coefficients adjusted for age group, sex, self-reported smoking and alcohol usage stratified by metabolic syndrome status.

Variables	MS participants										Non-MS participants									
	Mean 7-day METs	AEE (kcal/week)	TEE (kcal/week)	PAL	BMI (kg/m^2^)	WC (cm)	GGT (U/L)	TC (mmol /L)	TG (mmol/L)	WHtR	Mean 7-day METs	AEE (kcal/week)	TEE (kcal/week)	PAL	BMI (kg/m^2^)	WC (cm)	GGT (U/L)	TC (mmol/L)	TG (mmol/L)	WHtR
**Mean 7-day METs**																				
*r*	-	0.680*	0.970*	0.610*	−0.020	−0.010	−0.210	−0.210	−0.280*	−0.070	-	0.630*	0.570*	0.940*	0.090	0.050	−0.160**	−0. 070	−0.060	0.010
*p*	-	< 0.001	< 0.001	< 0.001	0.890	0.920	0.130	0.120	0.030	0.630	-	< 0.001	< 0.001	< 0.001	0.290	0.540	0.060	0.380	0.470	0.900
**AEE (kcal/week)**																				
*r*	0.680*	-	0.970*	0.610*	0.010	0.000	−0.200	−0.020	−0.220	0.010	0.630*	-	0.950*	0.600*	0.0020	−0.020	−0.150	0.040	−0.110	−0.060
*p*	< 0.001	-	< 0.001	< 0.001	0.930	0.890	0.140	0.890	0.100	0.930	< 0.001	-	< 0.001	< 0.001	0.980	0.840	0.080	0.670	0.180	0.450
**WC (cm)**																				
*r*	−0.010	0.020	0.120	0.010	0.930*	-	−0.090	−0.120	−0.180	0.950*	0.050	−0.020	0.030	0.110	0.870*	-	0.030	0.100	0.350*	0.960*
*p*	0.920	0.890	0.370	0.920	< 0.001	-	0.520	0.360	0.170	< 0.001	0.540	0.840	0.710	0.190	< 0.001	-	0.720	0.220	< 0.001	< 0.001
**GGT (U/L)**																				
*r*	−0.210	−0.200	−0.210	−0.160	−0.020	−0.090	-	0.170	0.13	0.050	−0.160	−0.150	−0.120	−0.050	0.020	0.030	-	0.090	0.190*	0.120
*p*	0.130	0.140	0.120	0.220	0.910	0.520	-	0.210	0.320	0.720	0.060**	0.080	0.130	0.540	0.780	0.720	-	0.270	0.020	0.150
**Chol (mmol/ L)**																				
*r*	−0.210	−0.020	−0.060	−0.140	−0.030	−0.120	0.170	-	0.330*	−0.020	−0.070	0.040	0.060	−0.050	0.100	0.100	0.090	-	0.280*	0.120
*p*	0.120	0.890	0.580	0.210	0.790	0.360	0.210	-	0.010	0.880	0.380	0.670	0.500	0.550	0.230	0.220	0.270	-	< 0.001	0.150
**TG (mmol/L)**																				
*r*	−0.280*	−0.220	−0.260*	−0.240**	−0.110	−0.180	0.130	0.330*	-	−0.090	−0.060	−0.110	−0.080	−0.040	0.230*	0.340*	0.190*	0.280*	-	0.360*
*p*	0.030	0.100	0.050	0.070	0.390	0.170	0.320	0.010	-	0.490	0.470	0.180	0.310	0.630	0.010	< 0.001	0.020	< 0.001	-	< 0.001
WHtR																				
*r*	−0.070	0.010	0.080	0.010	0.910*	0.950*	0.050	−0.020	−0.090	-	0.010	−0.060	−0.010	0.110	0.890*	0.960*	0.120	0.120	0.360*	-
*p*	0.630	0.930	0.540	0.970	< 0.001	< 0.001	0.720	0.880	0.490	-	0.900	0.450	0.930	0.190	< 0.001	< 0.001	0.150	0.150	< 0.001	-

AEE, activity energy expenditure; Chol, serum cholesterol; GGT, gamma-glutamyl transferase; MS, metabolic syndrome; PAL, physical activity level; TEE, total energy expenditure; TC, total cholesterol; TG, serum triglycerides; WC, waist circumference; WHtR, waist-to-height ratio.

* , The level of significance is set as *p* ≤ 0.05.

** , Borderline significance.

## Discussion

The study aimed to determine the relationship between PA, body fatness (i.e. WC, BMI and WHtR) and MS in urban South African teachers. In these relationships, WC was moderately and positively correlated with triglycerides in all the participants. Furthermore, our results showed a weak significant negative relationship between TEE and triglycerides amongst teachers classified with MS and an inconclusive weak borderline negative association between PAL and triglycerides. Also, a weak significant negative association between mean 7-day awake METs and triglycerides amongst the teachers classified with MS after adjustment for age group, sex, self-reported smoking and alcohol use was found.

The prevalence of MS was 29% in the total population, 46% in male teachers and 13% in female teachers according to the JIS for diagnosis of MS.^1^ Contradictory results have been reported in the US using data from the National Health and Nutrition Examination Survey (NHANES), indicating that nearly 35% of all adults and 50% of adults above the age of 60 years have MS.^32^ The observed prevalence of MS in our study is somewhat similar to 29% reported in urban participants from Gauteng Province,^8^ but when compared with the findings from an urban population in KwaZulu-Natal (26.5%), the prevalence of MS in the two former studies is observed to be high.^5^ The differences in the prevalence of MS may be because of varying criteria for the diagnosis of MS and the ethnic specificity of the WC cut-points used.^6^

The high prevalence of MS in this study may be because of the age of the participants (mean age in teachers with MS 51.25 ± 8.11 years vs. without MS 49.01 ± 8.50 years), as the prevalence of MS increases linearly with age.^4^ Another reason may be that all participants were classified as either sedentary (33%) or participated in light-intensity PA (67%), with 48% of the participants with MS being sedentary and 52% classified as lightly active. The risk of being diagnosed with MS increases to 73% when a person is regarded as physically inactive.^11,12^ In this study, an extremely high prevalence (92%) of abdominal obesity in persons with MS was observed, as indicated by the ethnicity-specific WC cut-point developed by Prinsloo and colleagues.^27^ Our findings, although inconclusive, suggest that the teachers with high abdominal fat could have influenced the high prevalence of MS.

The observed weak relationship between WC (i.e. JIS ethnic-specific cut-points clearly articulated WC as a contributor to MS^1^) and triglycerides in this study was similar to findings from the Family-Based Intervention Trial for Heart Health (FIT Heart) by Christian and co-workers,^33^ where it was reported that participants with increased WC were likely to have increased triglycerides. Furthermore, the observed relationship between WC and triglycerides confirms the well-known notion that visceral adipose tissue (VAT) is a key mediator, influencing glucose metabolism, BP, blood lipid and inflammatory profile, which are risk factors of metabolic syndrome.^34^ According to Tchernof,^35,36^ patients with increased VAT characteristically present with an increased level of low-density lipoprotein (LDL) cholesterol, which is associated with an increased fasting triglyceride level.^35^

In this study, a weak significant negative relationship between the PA variables (mean 7-day awake METs, TEE and AEE) and GGT in the total participants was found, indicating that participants who expend more energy have lower GGT levels. This result is in contradiction to a systematic review on PA and alcohol consumption that stated that higher rates of alcohol consumption were associated with higher levels of PA.^37^ A systematic review by Piazza-Gardner and Barry^38^ revealed that participants who were regular drinkers (self-reported alcohol consumption) participated in more PA than light or former drinkers. The contradictory results may be because four self-reported classifications (abstainers, former drinkers, light drinkers and drinkers) were used in the other study and in this study the exact number of drinks were not indicated to determine this association.^38^ Another reason for the results may be the significantly higher serum GGT in the teachers with MS than their non-MS counterparts in this study. The high prevalence of alcohol consumption may influence the incidence of MS, as heavy alcohol consumption is positively associated with the risk in developing MS whereas very light alcohol consumption is associated with a reduced risk of developing MS.^39^

However, after adjusting for age group, sex, self-reported smoking and alcohol consumption, this relationship in persons with MS was diminished but still existed in non-MS participants.

A weak significant negative relationship was found between triglycerides and mean 7-day awake METs, TEE and PAL; this is in contradiction with other studies on PA and dyslipidaemia that found no significant correlation between triglycerides and PA.^40,41^ Reasons for the disparity may be that in this study, the participants’ triglyceride levels were not classified as dyslipidaemia; however, a high prevalence of elevated triglycerides could have contributed to this result, indicating that participating in PA may be beneficial to triglyceride levels in people with MS. This relationship, however, was not present in the teachers who were not diagnosed with MS; the low prevalence of elevated triglycerides may be a possible explanation.

The strengths of the study were the inclusion of a unique group of teachers and highly standardised, objectively measured experimental protocols. A possible limitation to this substudy was the cross-sectional design, which may not infer causality. This study is limited by its sample size because of the strict adherence to the wearing time of the ActiHeart device. Therefore, the results cannot be generalised to the teachers of North West province or the entire population of South African teachers. Larger sample sizes and the inclusion of teachers from other provinces of South Africa would enable clinical comparisons. Another limitation of the study was that the individual step test calibration could not be performed because of the large number of participants in the larger study, the metabolic risk profiles of the participants and time constraints; however, a biokineticist (clinical exercise physiologist) thoroughly interviewed the participants about their weekly PA patterns before programming the ActiHeart device. Another limitation to consider in the interpretation of this findings is the dietary habits role in the observed relationships, which was not available during the analysis of current data. Given the significant role of diet to MS and PA, the authors recommend that future studies should include dietary patterns.

## Conclusion

There is an alarmingly high prevalence of MS amongst teachers in North West province of South Africa, as reflected by the prevalence of metabolic risk factors in the study participants. Most participants were classified as inactive or participating in light PA. The significant, although weak, relationship between mean 7-day awake METs and triglycerides in teachers with MS could indicate that PA may be beneficial in lowering triglyceride levels. Future studies should focus on developing adequate PA interventions for teachers to participate in and educating teachers about the risk factors that contribute to MS. Teachers should be made aware of the benefits of participating in regular PA.

## References

[1] Alberti KG, Eckel RH, Grundy SM, et al. Harmonizing the metabolic syndrome: A Joint Interim Statement of the International Diabetes Federation Task Force on Epidemiology And Prevention; National Heart, Lung, and Blood Institute; American Heart Association; World Heart Federation; International Atherosclerosis Society; and International Association for the Study of Obesity. Circulation. 2009;120(16):1640–1645. 10.1161/CIRCULATIONAHA.109.192644 19805654

[2] He D, Xi B, Xue J, Huai P, Zhang M, Li J. Association between leisure time physical activity and metabolic syndrome: A meta-analysis of prospective cohort studies. Endocrine. 2013;46:231–240. 10.1007/s12020-013-0110-0 24287790

[3] Mottillo S, Filion KB, Genest J, et al. The metabolic syndrome and cardiovascular risk: A systematic review and meta-analysis. JACC. 2010;56(14):1113–1132. 10.1016/j.jacc.2010.05.034 20863953

[4] Kirk EP, Klein S. Pathogenesis and pathophysiology of the cardiometabolic syndrome. J Clin Hypertens. 2009;11(12):761–765. 10.1111/j.1559-4572.2009.00054.x PMC2859214 20021538

[5] Motala AA, Mbanya JC, Ramaiya KL. Metabolic syndrome in sub-Saharan Africa. Ethn Dis. 2009;19:S2–S8. 19537344

[6] Okafor C. The metabolic syndrome in Africa: Current trends. Indian J Endocrinol Metab. 2012;16(1):56–66. 10.4103/2230-8210.91191 22276253 PMC3263198

[7] Van Zyl S, Van der Merwe LJ, Walsh CM, Groenewald AJ, Van Rooyen FC. Risk-factor profiles for chronic diseases of lifestyle and metabolic syndrome in an urban and rural setting in South Africa. Afr J Prim Health Care Fam Med. 2012;4(1):1–10. 10.4102/phcfm.v4i1.346

[8] George JA, Norris SA, Van Deventer HE, Crowther NJ. The association of 25 hydroxyvitamin D and parathyroid hormone with metabolic syndrome in two ethnic groups in South Africa. PLoS One. 2013;8(4):e61282. 10.1371/journal.pone.0061282 23596520 PMC3626636

[9] Grundy SM. Metabolic syndrome pandemic. Arterioscler Throm Vas. 2008;28(4):629–636. 10.1161/ATVBAHA.107.151092 18174459

[10] Camarillo-Romero E, Dominguez-Garcia MV, Amaya-Chavez A, et al. Effects of a physical activity program on markers of endothelial dysfunction, oxidative stress, and metabolic status in adolescents with metabolic syndrome. ISRN Endocrin. 2012;2012:970629. 10.5402/2012/970629 PMC3410314 22888450

[11] Edwardson CL, Gorely T, Davies MJ, et al. Association of sedentary behaviour with metabolic syndrome: A meta-analysis. PLoS One. 2012;7(4):e34916. 10.1371/journal.pone.0034916 22514690 PMC3325927

[12] Strasser B. Physical activity in obesity and metabolic syndrome. Ann N Y Acad Sci. 2013;1281(1):141–159. 10.1111/j.1749-6632.2012.06785.x 23167451 PMC3715111

[13] Bankoski A, Harris TB, McClain JJ, et al. Sedentary activity associated with metabolic syndrome independent of physical activity. Diabetes Care. 2011;34(2):497–503. 10.2337/dc10-0987 21270206 PMC3024375

[14] Baceviciene M, Luksiene D, Cesnaitiene V, Raubaite S, Peasey A, Tamosiunas A. Dose-response association between physical activity and metabolic syndrome. Open Med. 2013;8(2):273–282. 10.2478/s11536-012-0123-8

[15] Turi BC, Codogno JS, Fernandes RA, Monteiro HL. Low levels of physical activity and metabolic syndrome: Cross-sectional study in the Brazilian public health system. Cienc Saude Coletiva. 2016;21(4):1043–1050. 10.1590/1413-81232015214.23042015 27076003

[16] Scheers T, Philippaerts R, Lefevre J. SenseWear-determined physical activity and sedentary behavior and metabolic syndrome. Med Sci Sports Exerc. 2013;45(3):481–489. 10.1249/MSS.0b013e31827563ba 23034646

[17] Chu AH, Moy FM. Association between physical activity and metabolic syndrome among Malay adults in a developing country, Malaysia. J Sci Med Sport. 2014;17(2):195–200. 10.1016/j.jsams.2013.04.003 23665093

[18] Ainsworth BE, Haskell WL, Herrmann SD, et al. 2011 compendium of physical activities: A second update of codes and MET values. Med Sci Sports Exerc. 2011;43(8):1575–1581. 10.1249/MSS.0b013e31821ece12 21681120

[19] Laurence EC, Volmink J, Esterhuizen TM, Dalal S, Holmes MD. Risk of cardiovascular disease among teachers in Cape Town: Findings of the South African PaCT pilot study. S Afr Med J. 2016;106(10):996–1001. 10.7196/SAMJ.2016.v106i10.10869 27725020

[20] Malan L, Hamer M, Frasure-Smith N, Steyn HS, Malan NT. Cohort profile: Sympathetic activity and Ambulatory Blood Pressure in Africans (SABPA) prospective cohort study. Int J Epidemiol. 2015;44(6):1814–1822. 10.1093/ije/dyu199 25344943 PMC4689997

[21] Veldsman T, Swanepoel M, Monyeki MA, Brits JS, Malan L. Relationship between physical activity and carotid intima–media thickness among teachers in South Africa: The SABPA study. Cardiovasc J Afr. 2020;31(6):304. 10.5830/CVJA-2020-024 32716021

[22] Kohara K, Nishida W, Maguchi M, Hiwada K. Autonomic nervous function in non-dipper essential hypertensive subjects. Evaluation by power spectral analysis of heart rate variability. Hypertension (Dallas, TX: 1979). 1995;26(5):808–814. 10.1161/01.hyp.26.5.808 7591022

[23] Rogausch A, Kochen MM, Meineke C, Hennig J. Association between the BclI glucocorticoid receptor polymorphism and smoking in a sample of patients with obstructive airway disease. Addict Biol. 2007;12(1):93–99. 10.1111/j.1369-1600.2006.00045.x 17407502

[24] Sato N, Suzuki N, Sasaki A, et al. Corticotropin-releasing hormone receptor 1 gene variants in irritable bowel syndrome. PLoS One. 2012;7(9):e42450. 10.1371/journal.pone.0042450 22957021 PMC3434156

[25] Stewart A, Marfell-Jones M, Olds T, De Ridder JH. International standard for anthropometric assessment. 3rd ed. Wellington, New Zealand: International Society for the Advancement of kinanthropometry (ISAK); 2011, p. 137.

[26] Ashwell M, Hsieh SD. Six reasons why the waist-to-height ratio is a rapid and effective global indicator for health risks of obesity and how its use could simplify the international public health message on obesity. Int J Food Sci Nutr. 2005;56(5):303–307. 10.1080/09637480500195066 16236591

[27] Prinsloo J, Malan L, De Ridder JH, Potgieter JC, Steyn HS. Determining the waist circumference cut off which best predicts the metabolic syndrome components in urban Africans: The SABPA study. Exp Clin Endocrinol Diab. 2011;119(10):599–603. 10.1055/s-0031-1280801 22068551

[28] Assah FK, Ekelund U, Brage S, Wright A, Mbanya JC, Wareham NJ. Accuracy and validity of a combined heart rate and motion sensor for the measurement of free-living physical activity energy expenditure in adults in Cameroon. Int J Epidemiol. 2011;40(1):112–120. 10.1093/ije/dyq098 20529884

[29] Westerterp KR. Control of energy expenditure in humans. Eur J Clin Nutr. 2017;71(3):340–344. 10.1038/ejcn.2016.237 27901037

[30] Cohen JW. Statistical power analysis for behavioural sciences. 2nd ed. Hillsdale, NJ: Lawrence Erlbaum Associates; 1988.

[31] United Nations Department of International Economic and Social Affairs (UNDESA). Statistics: Provisional guidelines on standard international age classification of 1982. Statistics Papers Series M No 74. New York, NY: Publishing Service United Nations; 1982, p. 28.

[32] Aguila M, Bhuket T, Torres S, Liu B, Wong RJ. Prevalence of the metabolic syndrome in the United States, 2003–2012. JAMA. 2015;313(19):1973–1974. 10.1001/jama.2015.4260 25988468

[33] Christian AH, Mochari H, Mosca LJ. Waist circumference, body mass index, and their association with cardiometabolic and global risk. J Cardiometab Syndr. 2009;4(1):12–19. 10.1111/j.1559-4572.2008.00029.x 19245511 PMC2747723

[34] Despres JP, Lemieux I. Abdominal obesity and metabolic syndrome. Nature. 2006;444(7121):881–887. 17167477 10.1038/nature05488

[35] Tchernof A, Lamarche B, Prud ’Homme D, et al. The dense LDL phenotype: Association with plasma lipoprotein levels, visceral obesity, and hyperinsulinemia in men. Diabetes Care. 1996;19(6):629–637. 10.2337/diacare.19.6.629 8725863

[36] McNamara JR, Jenner JL, Li Z, Wilson PW, Schaefer EJ. Change in LDL particle size is associated with change in plasma triglyceride concentration. Arterioscler Thromb. 1992;12(11):1284–1290. 10.1161/01.ATV.12.11.1284 1420088

[37] Dodge T, Clarke P, Dwan R. The relationship between physical activity and alcohol use among adults in the United States: A systematic review of the literature. Am J Heal Promot. 2017;31(2):97–108. 10.1177/0890117116664710 27630108

[38] Piazza-Gardner AK, Barry AE. Examining physical activity levels and alcohol consumption: Are people who drink more active? Am J Health Promot. 2012;26(3):95–105. 10.4278/ajhp.100929-LIT-328. 22208422

[39] Sun K, Ren M, Liu D, Wang C, Yang C, Yan L. Alcohol consumption and risk of metabolic syndrome: A meta-analysis of prospective studies. Clin Nutr Elsevier Ltd. 2014;33(4):596–602. 10.1016/j.clnu.2013.10.003 24315622

[40] Chu AHY, Moy FM. Associations of occupational, transportation, household and leisure-time physical activity patterns with metabolic risk factors among middle-aged adults in a middle-income country. Prev Med (Baltim). 2013;57(Suppl):14–17. 10.1016/j.ypmed.2012.12.011 23276774

[41] Mohan V, Gokulakrishnan K, Deepa R, Shanthirani CS, Datta M. Association of physical inactivity with components of metabolic syndrome and coronary artery disease – The Chennai Urban Population Study (CUPS no. 15). Diabet Med. 2005;22(9):1206–1211. 10.1016/j.ypmed.2012.12.011 16108850

